# Supplementary material for: Development and Validation of the Weighted Index for Childhood Adverse Conditions (WICAC)
Source: Int J Environ Res Public Health. 2022 Oct 14;19(20):13251. doi: 10.3390/ijerph192013251 (PMC9602749; doi:10.3390/ijerph192013251)
Supplement: Supplementary file 1 [file ijerph-19-13251-s001.zip › ijerph-1903584-supplementary.pdf]

# Supplementary tables, figures and descriptions for the development and validation of the Weighted Index for Childhood Adverse Conditions

## Section 1: Development

### 1.1. Search Strategy II

With help from a librarian, we found useable MesH terms in PubMed to investigate which measurements were used. We used the following terms: “Reproducibility of Results” “Epidemiological Studies” “Surveys and Questionnaires” “Epidemiological Measurements” this was then combined with the exposure as “Adverse Childhood Experiences [MesH]”,” Childhood conditions”, “childhood terms”,” Growing up” and “upbringing”. The search was built in several narrower searches, when above a thousand hits, the first two hundred was included. The searches were conducted from February 2021 to April 2021. The strategy is shown in Fig. S1.

Inclusion criteria was: “measure descriptions”, “English or Scandinavian language”, and articles published after the included reviews (2016/18). The aim was to identify possible outcome measurements for our hypotheses and validation process. Therefore, the search was narrowed to include a somewhat homogeny population. We excluded specific population samples at high risk, such as LGBTQ+, and Afro-Americans, we further excluded specific countries, as to barriers in language, and at-risk populations, such as the Appalachian region in USA. We excluded duplicates, emerging in the included reviews to further secure a homogeny population.

## Fig. S1: Flow Chart, Literature search II

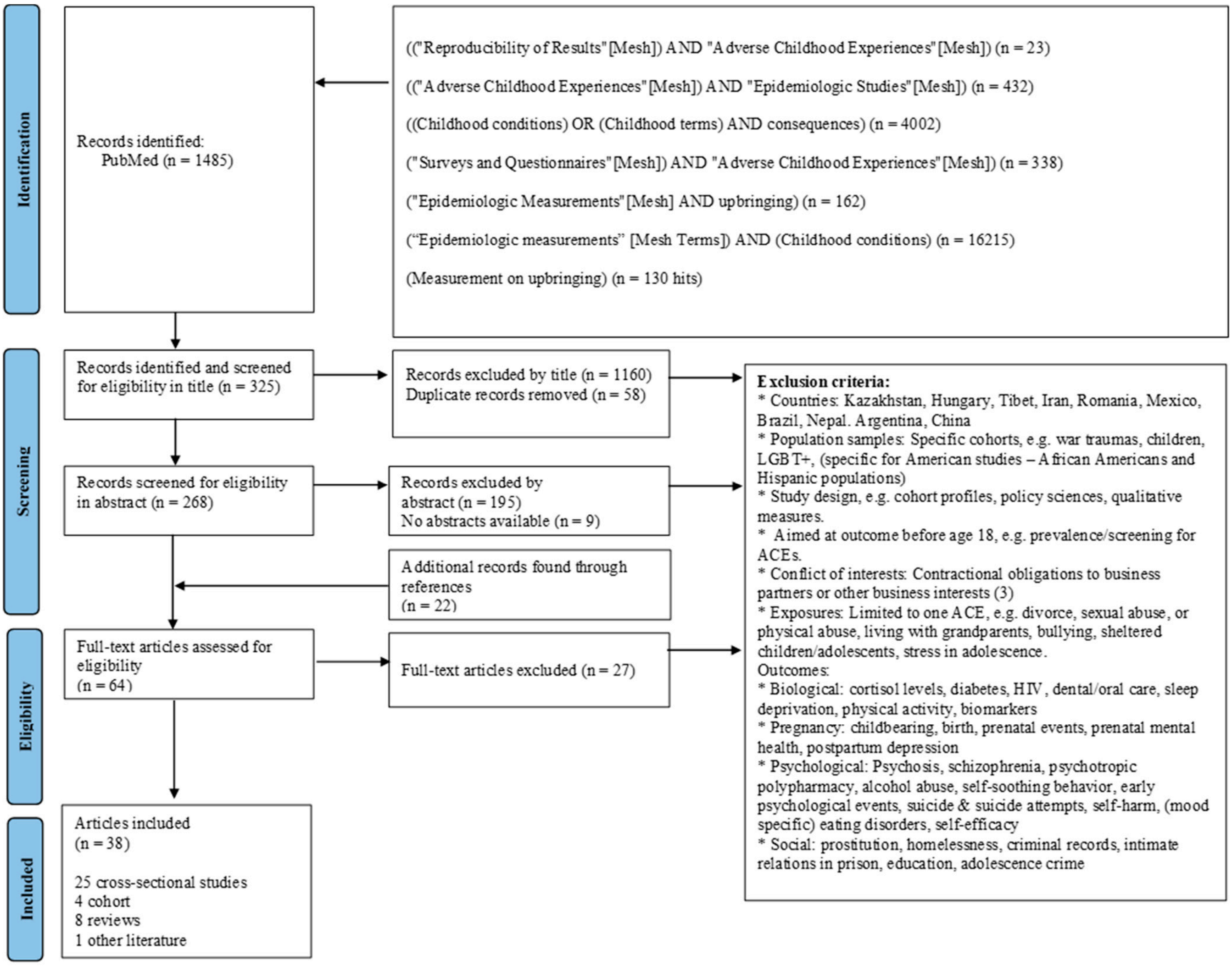


*Figure S1: The flow-chart describes the literature search for the theoretical development of WICAC*

### 1.2. Measure categories

Table S1 show the distribution of items included in the measures. The most common category, when measuring childhood adversities, is household dysfunction, followed by physical abuse, neglect, sexual abuse, emotional abuse, community factors and own injuries and health conditions, bereavement, loss and injury while only CAPA includes natural disasters as well. For other included items, some focus on categories such as  performance at school well-being among peers (CAPA (1), CCA (2), CLES (3), QUIC (4)) bullying (CAPA (1), CCA (2), JVQ-R2 (5), KID-SAVE (6) MACE (7)) and loneliness (CATS (8), CCA (2)). For the included categories, there were some variations in the form of the questions, and which questions were included. For household dysfunction there were items of parental substance abuse (ACE (9), CAPA (1), CATS (8), CCA (2)) parental mental illness (ACE (9),

CAPA (1), CCA (2)) witnessing violence against other family members e.g. siblings (CAPA (1), CATS (8), CCA (2), MACE (7)) and parental unemployment. (CCA (2), CLES (3))

According to own injuries and health conditions, some measures also included mental health and symptoms (CAPA (1), CCA (2)), while only 2 measures included unwanted, or teenage, pregnancy (CAPA (1), CLES (3))

While the CTQ-SF (10) and MACE (7) measures divided neglect into physical and emotional, QUIC (4) only measured emotional neglect. Only the JVQ-R2 (5) includes partner violence in physical abuse.

### Table S1: Categorical distribution of adversities according to included items in WICAC

| Table S1 | Categorical distribution of adversities according to the WICAC | | | | | | | | | |
|---|---|---|---|---|---|---|---|---|---|---|
| Measures | Adversities | | | | | | | | | |
| | Abuse | | | | | | | | | |
| | Physical | Sexual | Emotional | Neglect | Household Dysfunction | Community factors | Disasters | Bereavement, loss & injury | Own injuries and health | Other |
| ACE | ✓ | ✓ | ✓ | ✓ | ✓ | | | | | |
| CAPA | ✓ | ✓ | | | ✓ | ✓ | ✓ | ✓ | ✓ | ✓ |
| CATS | ✓ | ✓ | ✓ | ✓ | ✓ | | | | | ✓ |
| CCA | ✓ | | | | ✓ | ✓ | | | ✓ | ✓ |
| CLES | | | | | ✓ | | | ✓ | ✓ | ✓ |
| CTQ-SF | ✓ | ✓ | ✓ | ✓ | | | | | | |
| JVQ-R2 | ✓ | ✓ | ✓ | ✓ | ✓ | ✓ | | ✓ | | ✓ |
| KID-SAVE | | | | | ✓ | ✓ | | | ✓ | ✓ |
| MACE | ✓ | ✓ | ✓ | ✓ | ✓ | | | | | ✓ |
| QUIC | | | | ✓ | ✓ | | | | | ✓ |
| Table 3: Abbreviations: Adverse Childhood Experiences (ACE) Study, The Child and Adolescent Psychiatric Measure (CAPA), The Child Abuse and Trauma Scale (CATS), Cumulative Childhood Adversity (CCA), Cumulative Lifetime Adversity Measure (CLAM), Coddington Life Events Scale (CLES) Childhood Trauma Questionnaire, Short | | | | | | | | | | |

form (CTQ-SF), The Juvenile Victimization Questionnaire retrospective (JVQ-R2), KID-Screen for Adolescent Violence Exposure (KID-SAVE), The Maltreatment and Abuse Chronology of Exposure (MACE), The Questionnaire of Unpredictability in Childhood (QUIC), The Weighted Index for Childhood Adverse Conditions

### 1.3. Discussions on selection on weighting on each item

**1.3.1. Abuse:** In this category, we have included, physical abuse, sexual abuse, and emotional abuse. Contradicting the ACE-study (9), the items measuring physical abuse were divided to "Were physically attacked or insulted" and "Being physically harmed as a child (hit hard enough to leave a bruise or mark, kicked, burned etc.)". As studies suggest that being sexual abused is a higher risk factor for negative/poor health-related outcomes in adulthood than physical abuse (11, 12) we agreed to adjust the weight in the latter two parameters on the category of physical abuse to 2, as we defined a physical attack or assault as more common, e.g. the basic principle 2. Differently, from the ACE-study, we have included partner violence, as recommended by Afifi et al. 2020 (13).

The sexual abuse category is divided into what we can assume to be groping and rape. Studies suggest a minor difference in the severity and risk of these outcomes: We agreed the difference was not large enough to divide the weigh for the category, therefore we weighted factor 3 for all sexual abuse items, as all experts agreed that patients with a history of sexual abuse is often more trauma influenced.

For emotional abuse we included "Been shamed, embarrassed or told repeatedly that you are "no good", which is much like the item from the ACE-study. We further included "Been coerced with threats of harm to yourself or your family", as this item describes emotional stress both inside and outside the family. We weighted both items 2 according to estimates for emotional abuse in the literature, as well as to our 3. basic principal regarding "Been shamed,

embarrassed or told repeatedly that you are no good", as this item both can include bullying as well as a single experience of humiliation.

**1.3.2. Neglect:** We included the item "Were neglected (as a child) by your parent(s)" which is very much like the measure from the ACE-study and weights 2 according to existent literature (2), as well as our third basic principle as subjective experiences of neglect can vary. Further, we added "Experienced serious financial difficulties (i.e. no money for food or shelter)" as living in poverty is a very vital condition while growing up, as pointed out by Afifi et al. (2020). Further, an English study suggests that child-poverty is a major factor for adverse childhood experiences, as it can explain the health outcomes in some areas of England (14). However, as this was an ecological study, and as we discussed how poverty can be a general circumstance while growing up, we estimated the weigh for this item as 1.

**1.3.3. Household Dysfunctions:** Contradicting the ACE-study we included violence against both parents, and not just toward the motherly figure (9). We weighted this item 2, according to physical and emotional abuse. We included parents' divorce and weighted it 1 in line with Heidinger et al. (2020). Forced separation from family includes being in foster care, parental incarceration etc. as well as it can be very traumatic being removed from your family, we weighted the item 2 according to our third basic principle, as it can be a benefit for some children. Afifi et al. (2020) also recommends including items such as foster care and long-term separation from family due to deployment or immigration, which our item includes as well.

**1.3.4. Community Factors:** The ACE-study does not have any items describing community factors (9). As living in dangerous housing or neighborhood is closely associated with poverty, we weighted this item 1 as well. As for discrimination we weighted the item a factor 1 according to our second basic principle. As recommended by Afifi et al. (2020), we included items for witnessing and experiencing violence in the community. We weighted disaster 1, as

it is witnessed, and not experienced, the item for witnessed someone being injured or killed 2, according to our third basic principle.

**1.3.5. Disaster:** By including both natural disasters and war combat, we sought to cover the term of disaster, as a tragedy that man cannot control, in accordance with The Child and Adolescent Psychiatric Assessment (1). We weighted the item of natural disasters 2 according to our third basic principle, while we weighted combat in war a 3, considering children participating in combat.

**1.3.6. Bereavement, loss, and injuries:** Both parental death and death of a sibling are items recommended by Afifi et al. (2020), as experiencing a loss in your nearest family, or close friend, is proven to be a traumatic event, but not included in the terms of ACE, however, these events are definitely adverse conditions while growing up. Deaths by homicide or suicide and witnessed a family member being injured or killed rates 3, as we believe these events to be more traumatic than the loss under normal circumstances. Serious illness is rated 1, as it is not a deadly illness. Witnessed a family member being injured or killed rates to according to our third basic principle. The loss of a parent of sibling all rates 3, as these deaths influences the conditions while growing up in a more personal level than the death of a friend, which rates 2 according to the understanding of death among peers from a young age.

**1.3.7. Own injuries and health conditions:** Afifi et al. (2020), recommends including major childhood illness as an adversity in childhood, while teenage pregnancy has been found as being an adverse condition as well (1, 3). We discussed this category in reference to the purpose of the index, as if it would be used in research, we could adjust for some illnesses, which would introduce bias in the conducted research, however, we included the item, as it is an important condition for growing up, according to our construct. We rated unwanted pregnancy 1, as several members of the expert committee have experienced a lack of understanding and regret

in individuals who experienced an abortion at a young age.  We discussed that an unwanted pregnancy which resulted in a life born baby, also is dependable of other cultural dependencies which will show in other items as well. Suffering an illness or accident weighted a factor 2 conferring to Heidinger et al. (2019).

# 2. Validation

This section investigates additional descriptive analysis of missing values, descriptive analysis of WICAC according to specifics for all items and results for the sensitivity analyses.

## Fig. S2. Descriptive analysis of WICAC by adverse experiences

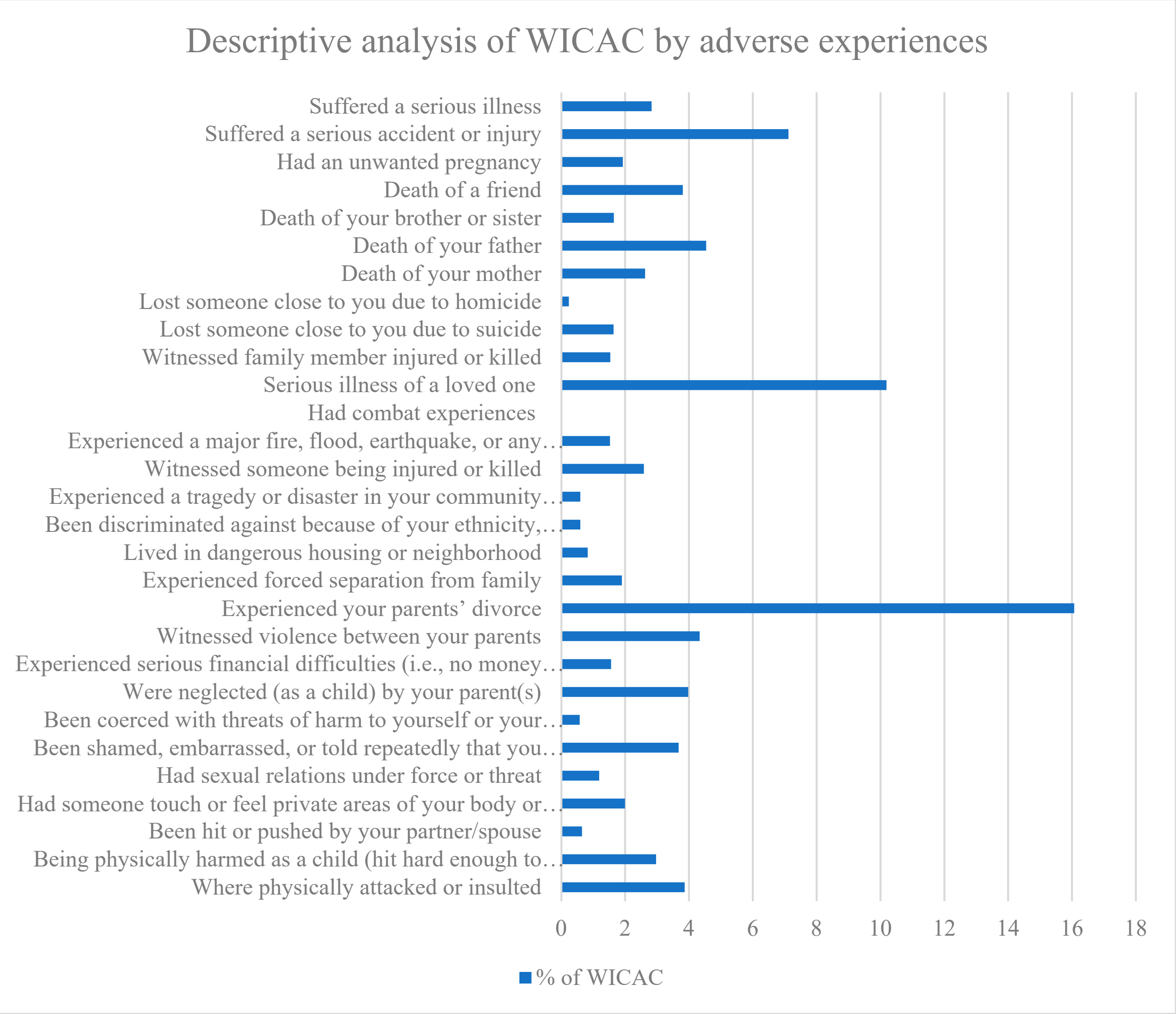


## Table S2: Descriptive analysis of WICAC by adverse experiences

| Tabel S2 | Descriptive analysis of WICAC by adverse experiences | | | |
|---|---|---|---|---|
| **Categories** | **Item** | **WICAC characteristics** | | |
| | | **% (N)** | **Median (IQR)** | **Min; Max** |
| ***WICAC*** | | ***84.88 (6360)*** | | ***0-46*** |
| **Abuse; Physical** | Where physically attacked or insulted | 3.87 (246) | 5.5 (2-11) | 1-46 |
| | Being physically harmed as a child (hit hard enough to leave a bruise or mark, kicked, burned etc.) | 2.97 (189) | 9 (5-16) | 2-46 |
| | Been hit or pushed by your partner/spouse | 0.65 (41) | 10 (5-14) | 2-30 |
| **Abuse; Sexual** | Had someone touch or feel private areas of your body or touched/felt anothers' private areas under force or threat | 2.00 (127) | 10 (6-18) | 3-43 |
| | Had sexual relations under force or threat | 1.19 (76) | 11 (6-23.5) | 3-43 |
| **Abuse; Emotional** | Been shamed, embarrassed, or told repeatedly that you are "no good" | 3.68 (234) | 9 (5-14) | 2-46 |
| | Been coerced with threats of harm to yourself or your family | 0.58 (37) | 11 (7-19) | 3-46 |

| | | | | |
|---|---|---|---|---|
| **Neglect** | Were neglected (as a child) by your parent(s) | 3.98 (253) | 10 (7-16) | 2-46 |
| | Experienced serious financial difficulties (i.e., no money for food or shelter) | 1.56 (99) | 7 (3-14) | 1-46 |
| **Household dysfunction** | Witnessed violence between your parents | 4.34 (276) | 7 (4-13) | 2-46 |
| | Experienced your parents' divorce | 16.07 (1022) | 3 (1-5) | 1-46 |
| | Experienced forced separation from family | 1.90 (121) | 8 (5-15) | 2-46 |
| **Community Factors** | Lived in dangerous housing or neighborhood | 0.83 (53) | 8 (4-14) | 1-46 |
| | Been discriminated against because of your ethnicity, religious background, or sexual orientation | 0.60 (38) | 6 (3-12) | 1-28 |
| | Experienced a tragedy or disaster in your community caused by people (a shooting, bombing, etc.) | 0.60 (39) | 7 (3-10) | 2-28 |
| | Witnessed someone being injured or killed | 2.59 (165) | 5 (2-8) | 2-32 |
| **Disaster** | Experienced a major fire, flood, earthquake, or any natural disaster in your community | 1.53 (97) | 4 (2-8) | 2-32 |
| | Had combat experiences | <5 | 6 (-) | 6-6 |
| **Bereavement, loss and injuries** | Serious illness of a loved one | 10.19 (648) | 3 (2-6) | 1-31 |
| | Witnessed family member injured or killed | 1.54 (98) | 6 (3-10) | 2-46 |
| | Lost someone close to you due to suicide | 1.64 (104) | 7 (4.5-9) | 3-46 |
| | Lost someone close to you due to homicide | 0.24 (15) | 8 (5-15) | 3-46 |
| | Death of your mother | 2.63 (167) | 5 (3-7) | 3-22 |
| | Death of your father | 4.54 (289) | 5 (3-7) | 3-33 |
| | Death of your brother or sister | 1.65 (105) | 5 (3-9) | 3-31 |
| | Death of a friend | 3.81 (242) | 4 (2-7) | 2-28 |
| **Own injuries and health conditions** | Had an unwanted pregnancy | 1.93 (123) | 3 (1-6) | 1-43 |
| | Suffered a serious accident or injury | 7.1 (453) | 3 (2-6) | 2-43 |
| | Suffered a serious illness | 2.83 (180) | 6 (4-8) | 2-30 |

Table S2: Abbreviations IQR=Interquartile range

## Table S3: Missing characteristics divided on index-categories

**Table S3** **Missing Characteristics divided in index-categories**

| **Variabel** | **Total WICAC (N:6360)** | **No Adversity (N:3383)** | **Low Adversity (N: 1293)** | **Moderate Adversity (N:1261)** | **Severe Adversity (291)** | **Very severe adversity (N: 132)** |
|---|---|---|---|---|---|---|
| Cardiovascular disease % (N) | 1.19 (76) | 1.4 (49)* | 0.8 (10)* | 0.7 (9) | 2.1 (6)* | 1.5 (2)* |
| Cancer % (N) | 1.43 (91) | 1.6 (55) | 1.1 (14) | 1.0 (13) | 2.4 (7)* | 1.5 (2)* |
| Poor Health % (N) | 0.46 (29) | 0.5 (17) | 0.3 (4) | 0.5 (6) | 0.7 (2) | - |
| Backpain % (N) | 0.80 (51) | 0.8 (28) | 0.9 (11) | 0.8 (10) | 0.7 (2) | - |

| | | | | | | |
|---|---|---|---|---|---|---|
| Obesity % (N) | | 0.1 (3) | - | 0.1 (1) | - | - |
| Anxiety % (N) | 1.29 (82) | 1.5 (51)* | 0.9 (11) | 0.9 (11) | 2.4 (7) | 1.5 (2) |
| Depression % (N) | 1.18 (75) | 1.4 (48) | 0.9 (11) | 0.7 (9) | 1.7 (5) | 1.5 (2) |
| Low vitality % (N) | 0.74 (47) | 0.8 (26) | 0.7 (9) | 0.6 (8) | 1.4 (4) | - |
| Low social status <4 % (N) | 0.97 (62) | 1.1 (38)* | 0.9 (11)* | 0.7 (9) | 1.4 (4)* | - |
| Low Education % (N) | | 0.3 (11) | 0.2 (3) | 0.2 (2) | 0.3 (1) | 0.8 (1) |
| Daily smoking % (N) | 0.27 (17) | 0.3 (9) | 0.3 (4) | 0.2 (3) | 0.3 (1) | - |
| Heavy drinking % (N) | 5.02 (319)* | 4.2 (141)* | 5.0 (65)* | 6.3 (80)* | 8.6 (25)* | 6.1 (8)* |
| Alcohol addiction % (N) | 5.17 (329)* | 4.3 (145)* | 5.2 (67)* | 6.5 (82)* | 8.9 (26)* | 6.8 (9)* |

Table S3: Heavy drinking is measured as >35 units pr. week. Addiction is measured as >1 on the CAGE-score.
* Increases the prevalence above 20 %.

# Table S4: RR & MD for WICAC and missing indices

| Table S4 | RR & Mean Differences between WICAC 50 % answered and >1 answered | | | | | | | | |
|---|---|---|---|---|---|---|---|---|---|
| Cases Index Categories | WICAC | | | 50 % answered | | | >= 1 answered | | |
| | RR/ MD | 95 % CI | P-Value | RR/ MD | 95 % CI | P-Value | RR/MD | 95 % CI | P-Value |
| ***Biological outcomes:*** | | | | | | | | | |
| Cardiovascular Disease*, RR<br>WICAC N=6104, 50 % Answered N=7023, >=1 answered N=7070 | | | | | | | | | |
| Low | 1.06 | (0.75-1.51) | 0.727 | 1.06 | (0.77-1.45) | 0.740 | 1.07 | (0.78-1.46) | 0.673 |
| Moderate | 0.90 | (0.64-1.27) | 0.545 | 0.98 | (0.72-1.32) | 0.884 | 0.96 | (0.71-1.30) | 0.800 |
| Severe | 1.65 | (0.89-2.68) | 0.072 | 1.57 | (0.94-2.61) | 0.084 | 1.56 | (0.94-2.59) | 0.089 |
| Very Severe | 1.09 | (0.42-2.82) | 0.864 | 0.92 | (0.35-2.40) | 0.868 | 0.92 | (0.35-2.40) | 0.866 |
| Cancer*, RR<br>WICAC N=6089, 50 % Answered N=7011, >=1 answered N=7059 | | | | | | | | | |
| Low | 1.05 | (0.81-1.36) | 0.714 | 1.05 | (0.83-1.32) | 0.711 | 1.04 | (0.83-1.32) | 0.714 |
| Moderate | 1.20 | (0.95-1.52) | 0.116 | 1.16 | (0.93-1.43) | 0.189 | 1.14 | (0.92-1.41) | 0.240 |
| Severe | 1.23 | (0.77-1.98) | 0.383 | 1.28 | (0.83-1.95) | 0.261 | 1.26 | (0.82-1.92) | 0.291 |
| Very Severe | 0.88 | (0.41-1.91) | 0.745 | 0.96 | (0.49-1.87) | 0.896 | 0.95 | (0.48-1.86) | 0.878 |
| Poor Health, RR<br>WICAC N=6153, 50 % Answered N=7088, >=1 answered N=7137 | | | | | | | | | |
| Low | 1.06 | (0.84-1.34) | 0.613 | 1.11 | (0.90-1.37) | 0.318 | 1.13 | (0.92-1.39) | 0.228 |
| Moderate | 1.44 | (1.20-1.72) | <0.0001 | 1.41 | (1.18-1.69) | <0.0001 | 1.43 | (1.20-1.71) | <0.0001 |
| Severe | 2.46 | (1.97-3.08) | <0.0001 | 2.51 | (1.98-3.17) | <0.0001 | 2.51 | (1.98-3.18) | <0.0001 |
| Very Severe | 2.16 | (1.83-2.91) | <0.0001 | 2.28 | (1.68-3.10) | <0.0001 | 2.31 | (1.70-3.12) | <0.0001 |
| Backpain, RR<br>WICAC N=6119, 50 % Answered N=7045, >=1 answered N=7093 | | | | | | | | | |
| Low | 1.13 | (1.01-1.26) | 0.036 | 1.10 | (1.00-1.22) | 0.057 | 1.11 | (1.00-1.23) | 0.042 |
| Moderate | 1.14 | (1.02-1.27) | 0.020 | 1.15 | (1.04-1.27) | 0.006 | 1.15 | (1.04-1.27) | 0.005 |
| Severe | 1.33 | (1.11-1.58) | 0.002 | 1.30 | (1.10-1.53) | 0.002 | 1.31 | (1.11-1.54) | 0.001 |
| Very Severe | 1.45 | (1.19-1.77) | >0.001 | 1.53 | (1.26-1.86) | <0.0001 | 1.54 | (1.27-1.86) | <0.0001 |
| BMI; kg/m2, MD<br>WICAC N=6164, 50 % Answered N=7100, >=1 answered N=7149 | | | | | | | | | |
| Low | 0.121 | (-0.176-(-0.417)) | 0.425 | 0.159 | (-0.117-0.436) | 0.258 | 0.146 | (-0.130-0.422) | 0.299 |
| Moderate | 0.506 | (0.209-0.803) | 0.001 | 0.453 | (0.176-0.731) | 0.001 | 0.437 | (0.161-0.713) | 0.002 |
| Severe | 0.765 | (0.210-1.320) | 0.007 | 0.761 | (0.246-1.276) | 0.004 | 0.771 | (0.257-1.285) | 0.003 |
| Very Severe | 0.628 | (-0.167-1.424) | 0.122 | 0.534 | (-0.190-1.259) | 0.148 | 0.523 | (-0.201-1.248) | 0.157 |
| Obesity, RR<br>WICAC N=6164, 50 % Answered N=7100, >=1 answered N=7149 | | | | | | | | | |

| | | | | | | | | | |
|---|---|---|---|---|---|---|---|---|---|
| Low | 1.06 | (0.91-1.23) | 0.444 | 1.09 | (0.95-1.25) | 0.210 | 1.09 | (0.95-1.25) | 0.195 |
| Moderate | 1.16 | (1.00-1.33) | 0.043 | 1.14 | (1.00-1.30) | 0.050 | 1.14 | (1.00-1.30) | 0.053 |
| Severe | 1.14 | (0.87-1.49) | 0.347 | 1.18 | (0.92-1.51) | 0.189 | 1.20 | (0.94-1.53) | 0.153 |
| Very Severe | 1.13 | (0.77-1.66) | 0.529 | 1.08 | (0.75-1.55) | 0.692 | 1.07 | (0.75-1.55) | 0.697 |
| ***Psychological outcomes*** | | | | | | | | | |
| Anxiety, RR<br>WICAC N=6103, 50 % Answered N=7025 >=1 answered N=7072 | | | | | | | | | |
| Low | 1.16 | (0.85-1.56) | 0.347 | 1.12 | (0.84-1.50) | 0.441 | 1.14 | (0.86-1.53) | 0.363 |
| Moderate | 1.15 | (0.86-1.54) | 0.353 | 1.21 | (0.91-1.59) | 0.187 | 1.22 | (0.92-1.61) | 0.162 |
| Severe | 2.49 | (1.70-3.66) | <0.0001 | 2.35 | (1.64-3.36) | <0.0001 | 2.35 | (1.64-3.37) | <0.0001 |
| Very Severe | 3.32 | (2.32-4.74) | <0.0001 | 3.01 | (2.06-4.41) | <0.0001 | 3.03 | (2.07-4.44) | <0.0001 |
| Depression, RR<br>WICAC N=6109, 50 % Answered N=7031 >=1 answered N=7078 | | | | | | | | | |
| Low | 1.23 | (1.03-1.47) | 0.021 | ***1.20 | (1.01-1.42) | 0.035 | ***1.21 | (1.02-1.43) | 0.027 |
| Moderate | 1.28 | (1.07-1.52) | 0.005 | ***1.37 | (1.17-1.61) | <0.0001 | ***1.40 | (1.19-1.64) | <0.0001 |
| Severe | 1.95 | (1.52-2.50) | <0.0001 | ***1.99 | (1.58-2.50) | <0.0001 | ***2.02 | (1.61-2.53) | <0.0001 |
| Very Severe | 2.49 | (1.97-3.13) | <0.0001 | ***2.82 | (2.35-3.40) | <0.0001 | ***2.85 | (2.37-3.43) | <0.0001 |
| Low Vitality, R<br>WICAC N=6136, 50 % Answered N=7067 >=1 answered N=7115 | | | | | | | | | |
| Low | 0.94 | (0.81-1.09) | 0.440 | 0.94 | (0.82-1.08) | 0.413 | 0.94 | (0.82-1.08) | 0.408 |
| Moderate | 1.06 | (0.93-1.22) | 0.381 | 1.11 | 0.98-1.26) | 0.106 | 1.11 | (0.98-1.26) | 0.103 |
| Severe | 1.40 | (1.14-1.71) | 0.001 | 1.56 | (1.31-1.86) | <0.0001 | 1.56 | (1.31-1.86) | <0.0001 |
| Very Severe | 1.75 | (1.40-2.17) | <0.0001 | 1.88 | (1.58-2.24) | <0.0001 | 1.88 | (1.57-2.24) | <0.0001 |
| ***Behavioral outcomes:*** | | | | | | | | | |
| Daily smoking, RR<br>WICAC N=6165, 50 % Answered N=7102, >=1 answered N=7151 | | | | | | | | | |
| Low | 1.08 | (1.01-1.16) | 0.026 | 1.08 | (1.01-1.15) | 0.016 | 1.08 | (1.02-1.15) | 0.013 |
| Moderate | 1.18 | (1.10-1.25) | <0.0001 | 1.17 | (1.11-1.24) | <0.0001 | 1.17 | (1.10-1.24) | <0.0001 |
| Severe | 1.28 | (1.15-2.42) | <0.0001 | 1.30 | (1.18-1.44) | <0.0001 | 1.30 | (1.18-1.43) | <0.0001 |
| Very Severe | 1.31 | (1.13-1.51) | <0.0001 | 1.34 | (1.17-1.52) | <0.0001 | 1.34 | (1.18-1.52) | <0.0001 |
| Smoking, amount, MD<br>WICAC N=2959, 50 % Answered N=3449, >=1 answered N=3471 | | | | | | | | | |
| Low | 0.247 | (-0.680-1.175) | 0.601 | 0.406 | (-0.449-1.262) | 0.352 | 0.387 | (-0.465-1.239) | 0.373 |
| Moderate | 1.904 | (1.014-2.793) | <0.0001 | 1.998 | (1.175-2.822) | <0.0001 | 2.049 | (1.229-2.868) | <0.0001 |
| Severe | 2.373 | (0.768-3.978) | 0.004 | 2.350 | (0.882-3.818) | 0.002 | 2.356 | (0.890-3.822) | 0.002 |
| Very Severe | 3.153 | (0.896-5.409) | 0.006 | 2.808 | (0.775-4.841) | 0.007 | 2.820 | (0.790-4.850) | 0.006 |
| Heavy Drinking, RR<br>WICAC N=5872, 50 % Answered N=6756, >=1 answered N=6797 | | | | | | | | | |
| Low | 1.11 | (0.64-1.89) | 0.716 | 1.08 | (0.65-1.80) | 0.758 | 1.08 | (0.65-1.79) | 0.765 |
| Moderate | 1.11 | (0.68-1.83) | 0.667 | 1.15 | (0.73-1.82) | 0.540 | 1.20 | (0.76-1.88) | 0.434 |
| Severe | 1.57 | (0.64-3.85) | 0.325 | 1.46 | (0.59-3.58) | 0.409 | 1.46 | (0.60-3.58) | 0.409 |
| Very Severe | 4.09 | (1.85-9.04) | <0.0001 | 4.36 | (2.07-9.10) | <0.0001 | 4.39 | (2.08-9.27) | <0.0001 |
| Alcohol addiction RR<br>WICAC N=5863, 50 % Answered N=6745, >=1 answered N=6789 | | | | | | | | | |
| Low | 0.93 | (0.70-1.24) | 0.613 | 0.96 | (0.74-1.25) | 0.762 | 0.96 | (0.73-1.25) | 0.743 |
| Moderate | 1.03 | (0.78-1.35) | 0.847 | 1.08 | (0.84-1.39) | 0.543 | 1.09 | (0.85-1.40) | 0.503 |
| Severe | 1.64 | (1.10-2.46) | 0.016 | 1.59 | (1.08-2.33) | 0.018 | 1.59 | (1.08-2.34) | 0.018 |
| Very Severe | 1.82 | (1.05-3.16) | 0.032 | 1.77 | (1.06-2.96) | 0.029 | 1.77 | (1.06-2.96) | 0.029 |
| Alcohol consumption, MD<br>WICAC N=5872, 50 % Answered N=6756, >=1 answered N=6797 | | | | | | | | | |
| Low | -0.124 | (-0.680-0.432) | 0.663 | -0.102 | (-0.623-0.419) | 0.701 | -0.106 | (-0.626-0.413) | 0.689 |
| Moderate | 0.309 | (-0.250-0.868) | 0.278 | 0.155 | (-0.369-0.680) | 0.561 | 0.195 | (-0.327-0.718) | 0.463 |
| Severe | 0.622 | (-0.437-1.680) | 0.250 | 0.342 | (-0.644-1.328) | 0.497 | 0.344 | (-0.642-1.330) | 0.494 |
| Very Severe | 1.606 | (0.103-3.109) | 0.036 | 1.053 | (-0.312-2.421) | 0.131 | 1.061 | (-0.306-2.428) | 0.128 |
| ***Social outcomes:*** | | | | | | | | | |
| Social Status**, MD<br>WICAC N=6297, 50 % Answered N=7271, >=1 answered N=7323 | | | | | | | | | |
| Low | -0.019 | (-0.109-0.071) | 0.680 | -0.028 | (-0.113-0.056) | 0.510 | -0.031 | (-0.115-0.054) | 0.478 |
| Moderate | -0.211 | (-0.301-(-0.121) | <0.0001 | -0.188 | (-0.273-(-0.103) | <0.0001 | -0.191 | (-0.275-(-0.106)) | <0.0001 |
| Severe | -0.353 | (-0.520-(-0.186) | <0.0001 | -0.381 | (-0.537-(-0.225)) | <0.0001 | -0.381 | (-0.537-(-0.225)) | <0.0001 |
| Very Severe | -0.481 | (-0.721-(-0.241) | <0.0001 | -0.520 | (-0.740-(-0.299)) | <0.0001 | -0.520 | (-0.740-(-0.299)) | <0.0001 |
| Low Social Status**, RR<br>WICAC N=6298, 50 % Answered N=7272, >=1 answered N=7224, | | | | | | | | | |
| Low | 1.24 | (0.80-1.91) | 0.337 | 1.05 | (0.70-1.58) | 0.807 | 0.98 | (0.64-1.49) | 0.914 |
| Moderate | 1.80 | (1.21-2.68) | 0.003 | 1.64 | (1.14-2.35) | 0.007 | 1.60 | (1.11-2.30) | 0.012 |

| | | | | | | | | | |
|---|---|---|---|---|---|---|---|---|---|
| Severe | 1.92 | (1.03-3.56) | 0.039 | 2.01 | (1.18-3.44) | 0.011 | 1.85 | (1.09-3.12) | 0.022 |
| Very Severe | 2.79 | (1.36-5.72) | 0.005 | 2.89 | (1.56-5.33) | 0.001 | 2.82 | (1.63-4.86) | <0.0001 |
| Low Education**, RR<br>WICAC N=6342, 50 % Answered N=7325 >=1 answered N=7378 | | | | | | | | | |
| Low | 1.09 | (0.98-1.20) | 0.097 | 1.08 | (0.98-1.19) | 0.094 | 1.08 | (0.98-1.19) | 0.102 |
| Moderate | 1.19 | (1.07-1.31) | 0.001 | 1.16 | (1.06-1.27) | 0.002 | 1.16 | (1.06-1.27) | 0.002 |
| Severe | 1.12 | (0.94-1.33) | 0.191 | 1.14 | (0.97-1.32) | 0.110 | 1.13 | (0.97-1.32) | 0.118 |
| Very Severe | 1.27 | (1.03-1.57) | 0.024 | 1.30 | (1.07-1.56) | 0.006 | 1.30 | (1.07-1.56) | 0.007 |
| ***Discriminative validation*** | | | | | | | | | |
| Handgrip strength, MD<br>WICAC N=6333, 50 % Answered N=7075, >=1 answered N=7124 | | | | | | | | | |
| Low | 0.081 | (-0.863-1.025) | 0.866 | -0.000 | (-0.888-0.889) | 0.999 | -0.012 | (-0.874-0.898) | 0.979 |
| Moderate | -0.458 | (-1.402-0.486) | 0.342 | -0.613 | (-1.505-0.278) | 0.177 | -0.645 | (-1.532-0.241) | 0.154 |
| Severe | -1.282 | (-3.048-0.485) | 0.155 | -1.495 | (-3.154-0.164) | 0.077 | -1.539 | (-3.194-0.116) | 0.068 |
| Very Severe | -2.108 | (-4.643-0.428) | 0.103 | -1.574 | (-3.906-0.757) | 0.186 | -1.575 | (-3.904-0.734) | 0.185 |

Table 9: Adjusted results: Age at inclusion, sex, social status, education. *Further adjusted for Smoking, BMI. **Only adjusted for age at inclusion and sex ***confounding variable for social status is categorized with 1-2,3,4,5,6,7,8,9,10 due to overfitting in analysis.
Abbreviations: RR: Risk Ratio, MD: Mean Difference in correlations, LA: Low Adversity, MA: Moderate Adversity, SA: Severe Adversity, VSA: Very Severe Adversity
*All RR and MD is according to reference group=No adversity.*

## Table S5: Cross-table of WICAC and an unweighted index

| **Unweighted Index** | **WICAC** | | | | | |
|---|---|---|---|---|---|---|
| | **No adversity** | **Low adversity** | **Moderate Adversity** | **Severe Adversity** | **Very Severe Adversity** | **Total** |
| **No adversity** | 3383 | - | - | - | - | 3383 |
| **Low adversity (1)** | - | 1206 | 358 | - | - | 1564 |
| **Moderate adversity (2-3)** | - | 87 | 836 | 112 | 3 | 1083 |
| **Severe adversity (4-6)** | - | - | 67 | 169 | 63 | 299 |
| **Very Severe adversity (>6)** | - | - | - | 10 | 66 | 76 |
| **Total** | 3383 | 1293 | 1261 | 291 | 132 | 6360 |

Table S5 shows the distribution on WICAC in the unweighted index

## Table S6: RR & MD for WICAC and the unweighted measure

| **Table S6** | **RR & Mean Differences between WICAC and an unweighted measure** | | | | | |
|---|---|---|---|---|---|---|
| **Cases** | **WICAC** | | | **Unweighted Index** | | |
| **Index Categories** | **RR/MD** | **95 % CI** | **P-Value** | **RR/MD** | **95 % CI** | **P-Value** |
| ***Biological outcomes:*** | | | | | | |
| Cardiovascular Disease*, RR, N=6104 | | | | | | |
| Low | 1.06 | (0.75-1.51) | 0.727 | 0.96 | (0.70-1.32) | 0.796 |
| Moderate | 0.90 | (0.64-1.27) | 0.545 | 1.08 | (0.74-1.57) | 0.689 |
| Severe | 1.65 | (0.89-2.68) | 0.072 | 1.26 | (0.67-2.38) | 0.471 |

| | | | | | | |
|---|---|---|---|---|---|---|
| Very Severe | 1.09 | (0.42-2.82) | 0.864 | 1.45 | (0.49-4.25) | 0.501 |
| Cancer*, RR, N=6089 | | | | | | |
| Low | 1.05 | (0.81-1.36) | 0.714 | 1.00 | (0.79-1.26) | 0.994 |
| Moderate | 1.20 | (0.95-1.52) | 0.116 | 1.32 | (1.02-1.70) | 0.036 |
| Severe | 1.23 | (0.77-1.98) | 0.383 | 1.33 | (0.85-2.07) | 0.220 |
| Very Severe | 0.88 | (0.41-1.91) | 0.745 | 0.94 | (0.32-2.74) | 0.921 |
| Poor Health, RR, N=6153 | | | | | | |
| Low | 1.06 | (0.84-1.34) | 0.613 | 1.13 | (0.92-1.39) | 0.240 |
| Moderate | 1.44 | (1.20-1.72) | <0.0001 | 1.57 | (1.28-1.93) | <0.0001 |
| Severe | 2.46 | (1.97-3.08) | <0.0001 | 2.13 | (1.62-2.79) | <0.0001 |
| Very Severe | 2.16 | (1.83-2.91) | <0.0001 | 2.39 | (1.51-3.78) | <0.0001 |
| Backpain, RR, N=6119 | | | | | | |
| Low | 1.13 | (1.01-1.26) | 0.036 | 1.15 | (1.04-1.28) | 0.006 |
| Moderate | 1.14 | (1.02-1.27) | 0.020 | 1.06 | (0.94-1.20) | 0.347 |
| Severe | 1.33 | (1.11-1.58) | 0.002 | 1.48 | (1.26-1.73) | <0.0001 |
| Very Severe | 1.45 | (1.19-1.77) | <0.001 | 1.57 | (1.20-2.05) | 0.001 |
| BMI; kg/m2, MD, N=6164 | | | | | | |
| Low | 0.121 | (-0.176-(-0.417)) | 0.425 | 0.173 | (-0.101-0.449) | 0.215 |
| Moderate | 0.506 | (0.209-0.803) | 0.001 | 0.485 | (0.163-0.807) | 0.003 |
| Severe | 0.765 | (0.210-1.320) | 0.007 | 1.131 | (0.585-1.677) | <0.0001 |
| Very Severe | 0.628 | (-0.167-1.424) | 0.122 | 0.084 | (-0.953-1.121) | 0.874 |
| Obesity, RR, N=6164 | | | | | | |
| Low | 1.06 | (0.91-1.23) | 0.444 | 1.06 | (0.92-1.21) | 0.440 |
| Moderate | 1.16 | (1.00-1.33) | 0.043 | 1.16 | (0.99-1.35) | 0.064 |
| Severe | 1.14 | (0.87-1.49) | 0.347 | 1.36 | (1.06-1.73) | 0.014 |
| Very Severe | 1.13 | (0.77-1.66) | 0.529 | 0.92 | (0.52-1.63) | 0.777 |
| ***Psychological outcomes:*** | | | | | | |
| Anxiety, RR, N=6103 | | | | | | |
| Low | 1.16 | (0.85-1.56) | 0.347 | 1.07 | (0.80-1.44) | 0.626 |
| Moderate | 1.15 | (0.86-1.54) | 0.353 | 1.34 | (0.99-1.81) | 0.060 |
| Severe | 2.49 | (1.70-3.66) | <0.0001 | 2.25 | (1.55-3.26) | <0.0001 |
| Very Severe | 3.32 | (2.32-4.74) | <0.0001 | 4.23 | (2.85-6.27) | <0.0001 |
| Depression, RR, N=6109 | | | | | | |
| Low | 1.23 | (1.03-1.47) | 0.021 | 1.18 | (0.99-1.39) | 0.059 |
| Moderate | 1.28 | (1.07-1.52) | 0.005 | 1.41 | (1.18-1.69) | <0.0001 |
| Severe | 1.95 | (1.52-2.50) | <0.0001 | 2.15 | (1.76-2.63) | <0.0001 |
| Very Severe | 2.49 | (1.97-3.13) | <0.0001 | 2.16 | (1.50-3.11) | <0.0001 |

| | | | | | | |
|---|---|---|---|---|---|---|
| Low Vitality, RR, N=6136 | | | | | | |
| Low | 0.94 | (0.81-1.09) | 0.440 | 0.94 | (0.82-1.08) | 0.375 |
| Moderate | 1.06 | (0.93-1.22) | 0.381 | 1.16 | (1.01-1.33) | 0.040 |
| Severe | 1.40 | (1.14-1.71) | 0.001 | 1.30 | (1.06-1.60) | 0.011 |
| Very Severe | 1.75 | (1.40-2.17) | <0.0001 | 1.87 | (1.45-2.41) | <0.0001 |
| Behavioral outcomes: | | | | | | |
| Daily smoking, RR, N=6165 | | | | | | |
| Low | 1.08 | (1.01-1.16) | 0.026 | 1.10 | (1.03-1.17) | 0.003 |
| Moderate | 1.18 | (1.10-1.25) | <0.0001 | 1.19 | (1.11-1.27) | <0.0001 |
| Severe | 1.28 | (1.15-2.42) | <0.0001 | 1.32 | (1.19-1.46) | <0.0001 |
| Very Severe | 1.31 | (1.13-1.51) | <0.0001 | 1.26 | (1.03-1.54) | 0.025 |
| Smoking, amount, MD, N=2959 | | | | | | |
| Low | 0.247 | (-0.680-1.175) | 0.601 | 0.886 | (0.038-1.733) | 0.041 |
| Moderate | 1.904 | (1.014-2.793) | <0.0001 | 1.604 | (0.629-2.581) | 0.001 |
| Severe | 2.373 | (0.768-3.978) | 0.004 | 2.680 | (1.106-4.254) | 0.001 |
| Very Severe | 3.153 | (0.896-5.409) | 0.006 | 2.124 | (-0.927-5.176) | 0.172 |
| Heavy Drinking, RR, N=5872 | | | | | | |
| Low | 1.11 | (0.64-1.89) | 0.716 | 1.28 | (0.81-2.02) | 0.283 |
| Moderate | 1.11 | (0.68-1.83) | 0.667 | 0.72 | (0.37-1.41) | 0.345 |
| Severe | 1.57 | (0.64-3.85) | 0.325 | 2.72 | (1.31-5.64) | 0.007 |
| Very Severe | 4.09 | (1.85-9.04) | <0.0001 | 3.98 | (1.56-10.17) | 0.004 |
| Alcohol Addiction, RR, N=5863 | | | | | | |
| Low | 0.93 | (0.70-1.24) | 0.613 | 0.97 | (0.74-1.25) | 0.790 |
| Moderate | 1.03 | (0.78-1.35) | 0.847 | 0.95 | (0.70-1.28) | 0.734 |
| Severe | 1.64 | (1.10-2.46) | 0.016 | 2.14 | (1.50-3.04) | <0.0001 |
| Very Severe | 1.82 | (1.05-3.16) | 0.032 | 1.26 | (0.54-2.96) | 0.590 |
| Alcohol consumption, units pr. week MD, N=5872 | | | | | | |
| Low | -0.124 | (-0.680-0.432) | 0.663 | 0.049 | (-0.468-0.565) | 0.854 |
| Moderate | 0.309 | (-0.250-0.868) | 0.278 | 0.011 | (-0.594-0.617) | 0.971 |
| Severe | 0.622 | (-0.437-1.680) | 0.250 | 1.553 | (0.522-2.584) | 0.003 |
| Very Severe | 1.606 | (0.103-3.109) | 0.036 | 1.389 | (-0.593-3.370) | 0.169 |
| ***Social outcomes:*** | | | | | | |
| Social Status** MD, N=6297 | | | | | | |
| Low | -0.019 | (-0.109-0.071) | 0.680 | -0.077 | (-0.160-0.007) | 0.072 |
| Moderate | -0.211 | (-0.301-(-0.121) | <0.0001 | -0.179 | (-0.277—0.081) | <0.0001 |
| Severe | -0.353 | (-0.520-(-0.186) | <0.0001 | -0.386 | (-0.551—0.221) | <0.0001 |
| Very Severe | -0.481 | (-0.721-(-0.241) | <0.0001 | -0.596 | (-0.909—0.282) | <0.0001 |

| Low Social Status**, RR, N=6298 | | | | | | |
|---|---|---|---|---|---|---|
| Low | 1.24 | (0.80-1.91) | 0.337 | 1.33 | (0.88-2.00) | 0.176 |
| Moderate | 1.80 | (1.21-2.68) | 0.003 | 1.84 | (1.22-2.77) | 0.004 |
| Severe | 1.92 | (1.03-3.56) | 0.039 | 1.92 | (1.05-3.50) | 0.033 |
| Very Severe | 2.79 | (1.36-5.72) | 0.005 | 2.71 | (1.11-6.60) | 0.028 |
| Low Education**, RR, N=6342 | | | | | | |
| Low | 1.09 | (0.98-1.20) | 0.097 | 1.11 | (1.01-1.23) | 0.027 |
| Moderate | 1.19 | (1.07-1.31) | 0.001 | 1.12 | (1.00-1.24) | 0.043 |
| Severe | 1.12 | (0.94-1.33) | 0.191 | 1.28 | (1.10-1.48) | 0.001 |
| Very Severe | 1.27 | (1.03-1.57) | 0.024 | 1.39 | (1.10-1.75) | 0.005 |
| ***Discriminative validation:*** | | | | | | |
| Handgrip strength, MD, N=6140 | | | | | | |
| Low | 0.081 | (-0.863-1.025) | 0.866 | -0.311 | (-1.187-0.564) | 0.486 |
| Moderate | -0.458 | (-1.402-0.486) | 0.342 | -0.077 | (-1.101-0.947) | 0.883 |
| Severe | -1.282 | (-3.048-0.485) | 0.155 | -0.856 | (-2.592-0.880) | 0.334 |
| Very Severe | -2.108 | (-4.643-0.428) | 0.103 | -3.789 | (-7.103—0.476) | 0.025 |

Table S6: Adjusted results: Age at inclusion, sex, social status, education. *Further adjusted for Smoking, BMI. **Only adjusted for age at inclusion and sex

Abbreviations: RR: Risk Ratio, MD: Mean Difference in correlations, LA: Low Adversity, MA: Moderate Adversity, SA: Severe Adversity, VSA: Very Severe Adversity

All RR and MD is according to reference group=No adversity.

## References


1. Angold A, Costello EJ. The Child and Adolescent Psychiatric Assessment (CAPA). Journal of the American Academy of Child and Adolescent Psychiatry. 2000;39(1):39-48.
2. Heidinger LS, Willson AE. The childhood roots of adult psychological distress: Interdisciplinary perspectives toward a better understanding of exposure to cumulative childhood adversity. Child abuse & neglect. 2019;97:104136.
3. Coddington RD. The significance of life events as etiologic factors in the diseases of children: I—A survey of professional workers. Journal of psychosomatic research. 1972;16(1):7-18.
4. Glynn LM, Stern HS, Howland MA, Risbrough VB, Baker DG, Nievergelt CM, et al. Measuring novel antecedents of mental illness: the Questionnaire of Unpredictability in Childhood. Neuropsychopharmacology : official publication of the American College of Neuropsychopharmacology. 2019;44(5):876-82.
5. Finkelhor D, Hamby S, Ormrod R, Turner H. The Juvenile Victimization Questionnaire: Reliability, validity, and national norms. Child abuse & neglect. 2005;29:383-412.
6. Flowers AL, Hastings TL, Kelley ML. Development of a Screening Instrument for Exposure to Violence in Children: The KID-SAVE. Journal of psychopathology and behavioral assessment. 2000;22(1):91-104.
7. Fosse R, Skjelstad DV, Schalinski I, Thekkumthala D, Elbert T, Aanondsen CM, et al. Measuring childhood maltreatment: Psychometric properties of the Norwegian version of the Maltreatment and Abuse Chronology of Exposure (MACE) scale. PloS one. 2020;15(2):e0229661.
8. Sanders B, Becker-Lausen E. The measurement of psychological maltreatment: Early data on the child abuse and trauma scale. Child abuse & neglect. 1995;19(3):315-23.
9. Felitti VJ, Anda RF, Nordenberg D, Williamson DF, Spitz AM, Edwards V, et al. Relationship of childhood abuse and household dysfunction to many of the leading causes of death in adults. The Adverse Childhood Experiences (ACE) Study. American journal of preventive medicine. 1998;14(4):245-58.
10. Bernstein DP, Stein JA, Newcomb MD, Walker E, Pogge D, Ahluvalia T, et al. Development and validation of a brief screening version of the Childhood Trauma Questionnaire. Child abuse & neglect. 2003;27(2):169-90.
11. Halpern CT, Tucker CM, Bengtson A, Kupper LL, McLean SA, Martin SL. Somatic symptoms among US adolescent females: associations with sexual and physical violence exposure. Maternal and child health journal. 2013;17(10):1951-60.
12. Kealy D, Rice SM, Ogrodniczuk JS, Spidel A. Childhood trauma and somatic symptoms among psychiatric outpatients: Investigating the role of shame and guilt. Psychiatry research. 2018;268:169-74.
13. Afifi T. Considerations for expanding the definition of ACEs. In: Asmundsen GJG, Afifi T, editors. Adverse childhood experiences : using evidence to advance research, practice, policy, and prevention. London, England: Academic Press; 2020. p. 35-46.
14. Lewer D, King E, Bramley G, Fitzpatrick S, Treanor MC, Maguire N, et al. The ACE Index: mapping childhood adversity in England. Journal of public health (Oxford, England). 2020;42(4):e487-e95.
